# Vitamin-D deficiency as a potential indicator of defective placentation in preeclampsia

**DOI:** 10.12669/pjms.40.11.9825

**Published:** 2024-12

**Authors:** Sanum Ali, Zilli Huma, Haleema Yasmin, Sehrish Hussain

**Affiliations:** 1 Sanum Ali, M.Phil. Anatomy Associate Professor, Department of Anatomy, Basic Medical Sciences Institute (BMSI), Jinnah Postgraduate Medical Centre (JPMC), Karachi, Pakistan; 2 Zilli Huma, FCPS (Gen. Surgery), PhD Anatomy Professor, Institute of Basic Medical Sciences, Khyber Medical University, Peshawar, Pakistan; 3 Haleema Yasmin, MBBS, MCPS, FCPS, MHPE Head of Gynecology and Obstetrics Units, Jinnah Postgraduate Medical Centre (JPMC), Karachi, Pakistan; 4 Sehrish Hussain, Department of Anatomy, Basic Medical Sciences Institute (BMSI), Jinnah Postgraduate Medical Centre (JPMC), Karachi, Pakistan

**Keywords:** Preeclampsia, Placenta, Vitamin-D_3_, Laminin, Immunohistochemistry

## Abstract

**Objective:**

To determine the association of serum Vitamin-D levels and placental laminin expression in pre-eclamptic and normotensive women.

**Methods:**

This cross-sectional study was conducted from July 2018 to February 2021, in the Department of Anatomy, Basic Medical Sciences Institute (BMSI) in collaboration with the Department of Gynecology and Obstetrics after the approval from Institutional Review Board (IRB), Jinnah Postgraduate Medical Centre JPMC, Karachi. The placentae were collected from 120 women, segregated into two cohorts as normotensive (NT) (n=60) and pre-eclamptic (PE) (n=60) and serum Vitamin-D levels measured. All placentae were examined for histological changes by Hematoxylin & Eosin stain and the expression of Laminin (LN) was calculated by the optical density using ImageJ.

**Results:**

The serum Vitamin-D levels (ng/ml) in normotensive were significantly higher as compared to pre-ecclamptics (p-value = 0.001). The Syncytial knots, cytotrophoblast proliferation and basement membrane thickness were also significantly different among normotensive and pre-eclamptics (p-value= 0.001)

When compared with normal placenta the optical density (expression) of LNs was significantly lower (p-value 0.001) in preeclampsia placentae. The association between serum Vitamin-D and laminin expression was highly positively significant. NT (r=0.811, p-value 0.001) and PE group (r=0.79, p-value 0.001)

**Conclusion:**

Our results show a strongly positive association between serum Vitamin-D levels, LN expression and severity of histopathological changes between normotensive and pre-eclamptic women.

## INTRODUCTION

Preeclampsia (PE) is a multifaceted disease specific to pregnancy, characterised by new onset of hypertension after 20 weeks of gestation and end-organ complications including proteinurea and dysfunctional cardiovascular, endocrine, and nervous system changes.^1^,^2^ Preeclampsia effects 1-7 % pregnancies worldwide and may lead to severe maternal and neonatal morbidity and mortality.^3^ The aetiology and pathophysiology of preeclampsia is complex and various maternal and foetal determinants along with macro and micronutrient deficiencies have been proposed for its pathogenesis.^4^ In essence, preeclampsia is a disorder of the placenta, and most theories revolve around shallow trophoblast invasion and abnormal placentation.^2^

Vitamin-D is a key hormone and important nutrient that plays a crucial role in the regulation of calcium homeostasis and bone metabolism as its classical effects.^5^ It also has non-classical biological effects in almost every tissue of the body including cell proliferation and adherence, differentiation, apoptosis and immune function.^6^,^7^ Role of Vitamin-D as a trace element in cellular and numerous biochemical pathways is well studied and alterations in Vitamin-D homeostasis may lead to pathogenesis of many disorders of pregnancy as the likelihood of developing nutritional deficiencies (macro and micronutrients) is higher in pregnant women because of the increased demands of developing fetus.^8^,^9^ Evidence from recent epidemiological research has shown a link between maternal Vitamin-D deficiencies with pathogenesis of preeclampsia.^10^

Continuing research on better understanding of the aetiology of preeclampsia has identified many possible targets at molecular level as predictive and likely measures for treatment of preeclampsia. One of the crucial molecules that might play a central role in both normal and pathological trophoblast invasion is Laminin (LN), a glycoprotein that forms an integral part of basement membrane (BM).^11^ Primarily its role lies in villous organization by physically outlining the epithelial and endothelial cells and promoting cell growth and tethering the trophoblast and endothelial cells to the villous core.^12^ Any alterations in the structure of BM may affect the villous perfusion. Thickening of placental Basement membrane is seen in many placental diseases including preeclampsia.^13^ Various studies have laid out an association between low serum levels of Vitamin-D and pathological changes in mucosal proteins and ECM.^14^ Thus, this study was designed to identify an association between serum Vitamin-D levels and laminin expression in patients with preeclampsia and normotensive pregnant women.

## METHODS

This study was conducted from July 2018 to February 2021 in the department of Anatomy, Basic Medical Sciences Institute (BMSI), in collaboration with the Department of Obstetrics and Gynecology, Jinnah Postgraduate Medical Centre, Karachi. The sample size of 84 was calculated by using the open epi mean difference formula with 95% confidence interval. The mean value of Pre-eclamptic group was taken as 6.78 ± 3.55 and Normotensive group was taken as 9.43 ± 4.86. The sample size enhanced to 60 in each group to get more statistical precise difference of two mean.^15^ These pregnant women in their third trimester were recruited from antenatal clinic by non-probability purposive sampling with normotensive (NT) (n=60) and PE (n=60).

### Ethical Approval:

This cross-sectional comparative study was conducted after seeking approval of the Institutional Review Board (IRB) (NO.F.2-81-IRB/2018-GENL/816/JPMC; dated December 16, 2021).

### Inclusion criteria:

Pregnant ladies with gestational age >32 weeks and maternal age >20 years were included in this study. In case of the PE group the American College of Obstetricians and Gynecology (ACOG) criteria 2013 was used.^16^

### Exclusion criteria:

Any participant with any other known chronic systemic disease, i.e., cardiovascular, urogenital, immunological or endocrinological were excluded.

Peripheral blood (5ml) was drawn from the respective females and dispensed in a vacutainer containing EDTA as anti-coagulant. Blood was stored at -20^o^C and later thawed at 4^o^C gradually, one day prior to use for serum Vitamin-D analysis. The serum Vitamin-D was estimated according to user’s manual guide, using *ELISA kit* from DIA Source (250H Vitamin-D Total Elisa 90), Immunoassays (S.A. Rue du Bosquet, DIA, 2-B-1348 Louvain-la-Neuve- Belgium). Tests were run in triplicates. The analysis of Vitamin-D status included 25(OH) Vitamin-D (25(OH)D) levels in maternal blood (Sufficiency >30 ng/ml, insufficiency 21-29 ng/ml, deficiency <20 ng/ml (According to Agha Khan medical university laboratories (AKU) clinical laboratories institution standard guidelines.^17^

Placenta from the recruited subjects were collected after delivery (caesarean section/ spontaneous vaginal delivery) and stored in 10% Buffer Neutralized Formalin (BNF). Paraffin embedded block of the placental tissues was made after conventional fixation. The blocks were stored in laboratory with designated code. Sections of 5μm were stained with hematoxylin & eosin (HE) and periodic acid Schiff (PAS). The cytotrophoblast proliferation (CT) (/100 villi)^2^, syncytial knots (SK) (/100 villi)^2^ and basement membrane thickness (μm) > 20 percent terminal villi^20^ were measured in Fiji imageJ software. The investigator was blinded while analysing the morphological changes.

Expression of Laminin was explored by immunohistochemistry by probing the 5μm thick sections from the paraffin embedded blocks of placentae with laminin polyclonal antibody (diluted at 1:100) (Thermo-Fisher Scientific PA1-711). Immunohistochemical analysis of the blocks was done after antigen retrieval and blocking with Bovine Serum Albumin. 4) This was followed by incubation with anti-rat primary laminin antibody diluted at (1:100), Horse Raddish peroxidase (HRP), conjugated secondary antibodies and DAB (3,3’-diaminobenzidine tetrahydrocholoride) chromogen with counterstain by hematoxylin. Microscopic images were taken by light microscope (Nikon Eclipse 5oi; Japan) connected to video link digitalizing board system (DS Camera control unit- DS-L2) and analysed on Fiji imagej software for analyzing the optical density (OD) = log (max intensity/Mean intensity), where max intensity = 255 for 8-bit images.

### Statistical analysis:

The data was analyzed on SPSS version 23.0. The comparison of mean values of quantitative variables among groups was done by independent sample t-test and for association among groups within variables correlation coefficient (r) was computed. The intensity of the expression (optical density) was quantified using image J IHC profiler. The results were considered significant at p≤0.05.

## RESULTS

Demographic variables of the two groups of 60 pre-eclamptic (PE) and 60 normotensive (NT) pregnant ladies are shown in Table-I. The PE group had a significantly greater gestational age but a lower BMI as compared to the NT group. There was hardly any significant difference in the mean ages of the two groups (0.99±2.174).

**Table-I:** Comparison of demographic variables in Pre-eclamptic versus Normotensive Groups.

Variables	Groups	n	Mean	Std. Deviation	p-value
Age (Years)	Pre-Eclamptic	60	27.42	3.581	0.05
	Normal	60	26.43	1.407	
Gestational age (Weeks)	Pre-Eclamptic	60	37.53	2.111	0.001
	Normal	60	39.07	0.312	
BMI (kg/m2)	Pre-Eclamptic	60	26.16	1.196	0.001
	Normal	60	27.04	1.138	
	Normal	11	17.27	2.4286	

Most of the Preeclampsia group, (78.3%) had severe hypertension (≥ 160/ ≥ 110 mmHg) with a significant increase in blood pressure of preeclampsia as compared to normotensive group, p-value= 0.001 (independent sample t-test) (Table-II).

**Table-II:** Severity of Blood pressure in Pre-eclamptic versus Normotensive Groups.

Variables	Groups	n	Mean	Std. Deviation	p-value
Systolic BP at booking (mmHg)	Pre-Eclamptic	60	164.33	17.307	0.001
	Normal	60	124.67	7.003	
Diastolic BP at booking (mmHg)	Pre-Eclamptic	60	96.33	6.881	0.001
	Normal	60	80.92	2.836
***Subdivision on the Basis of Severity of BP***					
*BP (mmHg) Groups (SBP/DBP)*	*Pre-eclamptic*	*p-value*			
Mild	13	0.001			
Severe	47				
Total	60				

* Mild ≥140/(≥90) and 160/90mmHg *Severe (≥160/(≥110) mmHg, P-value Student T test.

On the basis of serum Vitamin-D3 level subjects were divided into three groups (Table-III), sufficient, insufficient and deficient. The mean Vitamin-D3 levels in the PE group were significantly less than the normotensive group (p-value 0.001), 18.14 ± 5.67ng/ml and 26.42 ± 6.14 ng/ml, respectively. In the normotensive group most of the patients had insufficient or deficient Vitamin-D3 levels, 78.3% compared to 95% of the pre-eclamptic patients. Overall comparison of Vitamin-D3 levels between the two groups is highly significant p-value 0.001, 2-sample *t* (Table-III).

**Table-III:** Serum Vitamin-D3 levels in Pre-eclamptic versus Normotensive Groups.

Sub-Groups Serum Vit D3 (ng/ml)					

	Groups	n	Mean	Std. Deviation	p-value
Sufficient	Pre-Eclamptic	3	30.93	0.7371	0.368
	Normal	23	32.48	2.8498	
Insufficient	Pre-Eclamptic	22	22.37	1.8767	0.001
	Normal	26	24.94	2.5809	
Deficient	Pre-Eclamptic	35	14.38	3.6114	0.017
	Normal	11	17.27	2.4286	

**Fig.1:**
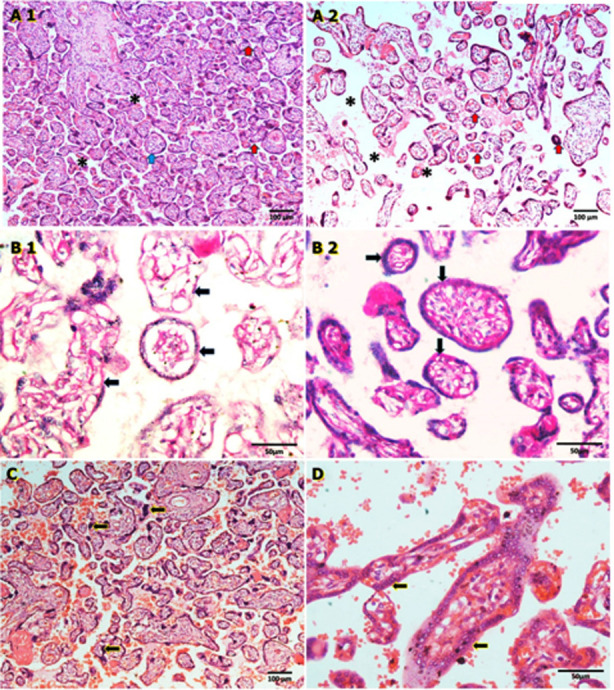
Microscopic features in Normotensive (A1, B1) and Pre-eclamptic (A2, B2, C, D). A1: Normal placenta with intervillous space (asterix) Blue arrow mature villi. A2: Pre-eclamptic with reduced villous density (asterix), 100X B1: Normal placenta intact villous B2: Pre-eclamptic with thickened basement membrane. Black arrows, 400X C: Pre-eclamptic with syncytial knots. Black arrows, 100X D: Pre-eclamptic with Cytotrophoblastic proliferation. Black arrows, 400X.

Microscopic examination exhibited a statistically significant increase in syncytial knots, cytotrophoblast proliferation and basement membrane thickness in PE group compared to control group (p-value 0.001, 2-sample t) (Table-IV). The mean difference in the thickness of the basement membrane between the two groups was 14.45μm. Similarly, the cytotrophoblast proliferation in cases was higher with a mean difference of 1.14 as compared to the control group. The syncytial knots were also increased in pre-eclamptics as compared to the control group with a significant difference of 21.17 between the groups (Fig.1, Table-IV).

**Table-IV:** Pearson Correlation of Optical Density of Laminin and histological features with serum Vitamin-D3 (ng/ml) of the two groups (Pre-eclamptic and Normotensive).

	Groups	n	Mean	Std. Deviation	t-value	p-value	Correlation Coefficient "r"
Optical Density Laminin (IHC)	Pre-Eclamptic	60	0.119	0.064	6.33	0.001	r= 0.79 p=0.001
	Normal	60	0.198	0.073			r= 0.811 p=0.001
Syncitial Knots	Pre-Eclamptic	60	28.210	2.334	65.01	0.001	r= 0.058 p=0.658 r=-0.432 p=0.008*
	Normal	60	7.045	0.956			
Cytotrophoblast proliferation	Pre-Eclamptic	60	2.435	0.325	23.75	0.001	r= -0.093 p=0.478 r=-0.575 p=.001*
	Normal	60	1.292	0.183			
Basement Membrane Thickness (μm)	Pre-Eclamptic	60	28.16	0.639	181.58	0.001	r= 0.309 p= 0.062
	Normal	60	13.71	0.220			r= -0.209 p= 0.106

**Fig.2:**
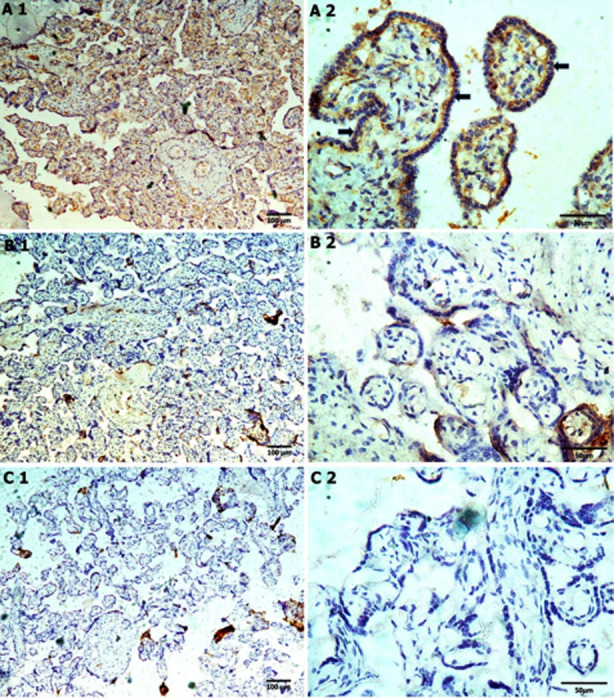
Immunohistochemical expression of Laminin in Normal (A1, A2), Mild (B1, B2) and Severe (C1, C2) preeclampsia. A1, B1, C1 (100X), A2, B2, C2 (400X) Black arrows Laminin expression.

Immuno-histochemical examination showed the homogenous distribution of laminin expression in the basement membrane of chorionic villi and fetal capillaries in normotensive as compared to pre-eclamptics (Fig.2 A1, B1). Laminin expression decreased further with severity of preeclampsia (Fig.2 C,D). The optical density of laminin was significantly reduced in pre-eclamptics to 0.119 ± 0.64 as compared to 0.198 ± 0.73 in normotensives, p-value 0.001 (Table-IV). The histological features in the PE group were all significantly increased as compared to the NT group.

A Pearson correlation coefficient was computed to assess the linear relationship between Serum Vit D3 levels and laminin expression with a positive correlation in both groups, of 0.79 and corresponding p-value of 0.001in pre-eclamptic group. There was a moderately negative correlation with Synctial knots and cytotrophoblastic proliferation of .43 and -.57, respectively with corresponding p-values of 0.008 and 0.001, respectively (Table-IV).

## DISCUSSION

In this study the serum Vitamin-D levels (ng/ml) in pre-eclamptics were significantly reduced as compared to normotensives. When compared with normal placenta laminin expression was significantly reduced in pre-eclamptic placentae. There histomorphological parameters as; syncytial knots, cytotrophoblast proliferation and basement membrane thickness were significantly higher in pre-eclamptics. There have been numerous studies on the cause and effect of pre-eclampsia on perinatal morbidity and mortality. In this regard the placenta may be considered a reflection of perinatal mortality. Studies have revealed that maternal diseases like diabetes mellitus, preeclampsia and eclampsia exert deleterious effects on the placenta. Pre-eclampsia effects 1-7% pregnancies and may lead to serious maternal and perinatal morbidity.^3^

In addition to these any nutritional deficiencies have further adverse effects on pregnancy outcomes. Hypovitaminosis D is becoming a serious health problem, effecting approximately one billion people worldwide.^15^ Nutritional, micronutrient (vitamin) deficiencies and unfortified food is recently linked to play a role in the development of preeclampsia. Anomalistic placentation is a key feature of pre-eclampsia that link to altered placental histopathological features.^8^,^15^ In our study, serum Vitamin-D3 levels were investigated to find any potential role of Vitamin-D in defective placentation in pre-eclamptics.

In this study, 120 pregnant women were inducted, 60 normotensive as control group and 60 with diagnosed preeclampsia (according to blood pressure estimation and degree of proteinurea). Maternal age was comparable in both groups. Gestational age and basal metabolic rates (BMI) were statistically different in both groups. Our findings are comparable to a previous study by Salam and colleagues who have linked high pre-pregnancy BMI to various hypertensive disorders of pregnancy.^18^ Our study revealed that there was a statistically significant relation between serum Vitamin-D levels and blood pressure. These results are consistent with Mohammad et al, who found a direct relationship between severity of preeclampsia and severe deficiency of Vitamin-D.^19^

On microscopic level, our results showed that histological features like villous Basement membrane thickness, Cytotrophoblast proliferation, Syncitial Knots and Laminin expression in the villous Basement membrane were significantly altered in placenta from Preeclampsia cases in comparison to placenta from normal term pregnancy. These notable microscopic changes signify the structural adaptive mechanism for placental ischemia in hypertension. Research by Zhang et al., suggests a complex interplay of the combination of low serum Vitamin-D levels & low Laminin expression in preeclampsia that may further intensify placental morphological alterations, possibly contributing to complications in maternal health and fetal development by impacting the placenta’s structural integrity and function during pregnancy.^13^ Our findings stand by well with other study which showed that defective placental morphology may lead to impaired placental perfusion, which is the hallmark of Preeclampsia and the histological malformation was confirmed on Doppler velocimetry.^20^

Excessive Syncitial Knots and Cytotrophoblast proliferation in preeclampsia is an adaptive approach of placenta to insufficient utero-placental blood flow. Reduced blood flow to syncytium due to impaired perfusion of intervillous space may cause syncytial hypoxial damage.^21^ This damage leads to a compensatory syncytial nuclear proliferation and may cause excessive formation of Syncitial Knots. In order to mend the damaged syncytium, Cytotrophoblast cells proliferate immoderately; this occurrence may explain the higher number of syncytial knots and Cytotrophoblast proliferation in our study.^20^ We observed denuded BM of trophoblast at the site of thickening. Thickening of Basement membrane is a result of mucopolysaccharide accumulation which could be attributed to Cytotrophoblast proliferation, since these cells secret villous Basement membrane proteins, excessive proliferation may lead to abnormal amount of Basement membrane proteins in ischemic placenta in preeclampsia and Diabetes Mellitus.^22^

We analysed the relationship between Laminins and preeclampsia from the viewpoint of varied expression of Laminin in basement membrane of villous trophoblasts. Our results revealed that Laminin expression was decreased in preeclampsia, and further comparison between mild and severe preeclampsia groups showed that Laminin was profoundly decreased in severe group than mild (Fig.2). Laminins (LNs) are an essential family of extracellular matrix (ECM) molecules that make up the Basement membrane and furnish a distinctive milieu for spatial and molecular information to synchronize and regulate implantation and placentation.^23^

Interaction of Laminin with cell surface receptors triggers intracellular signals to initiate cell adhesion and angiogenesis. Successful trophoblastic invasion of the uterine decidua and maternal vascular system eventually leads to embryo implantation.^10^ Recently Aplin and colleagues (2020) distinctly summarized the steps in normal and failed uterine spiral arterial conversion due to defective spiral arteries remodeling, hence impaired implantation, which could be the pathological and clinical consequence of preeclampsia.^24^ One of the non-classical roles of Vitamin-D is its effect and regulation of tight junctional proteins of mucosa and Basement membrane which may explain its defective morphology in Vitamin-D deficiency.^25^

Hence our results have shown a strongly positive association between serum Vitamin-D levels and optical density of laminin leading to defective placentation in pre-eclamptic women, thus the inclusion of Vitamin-D supplementation may be started in the antenatal period which can contribute to better feto-maternal outcome.

### Limitations and recommendations:

Lack of financial resources could not allow us to expand our work as a multicenter study. Further studies with other gene markers and a bigger sample size can provide more insight on the pathophysiology of preeclampsia.

## CONCLUSION

Our work showed an overall trend of hypovitaminosis D in control and cases; however, it was more statistically significant in preeclampsia cases and was associated with more destructive histological features in adaptation to placental insufficiency.
